# Neuropsychological Correlates of Anxiety in Older Adults: A Cross-Sectional Comparison with Controls Without a Psychiatric Diagnosis

**DOI:** 10.3390/brainsci16090949

**Published:** 2026-09-05

**Authors:** Yasemin Koçyiğit, Ayşe Gökçen Gündoğmuş, Cansu Çoban, Metehan Bitlisli, Şule Bıçakcı Ay, Özlem Erden Aki

**Affiliations:** 1 Department of Psychiatry, Ankara Etlik City Hospital, Ankara 06170, Turkey; drysmnkcygt@hotmail.com (Y.K.); gokcengonen@gmail.com (A.G.G.); cansusen.autf@gmail.com (C.Ç.); psk.metehanbitlisli@gmail.com (M.B.); 2 Department of Psychiatry, Hacettepe University, Ankara 06230, Turkey; ozlem.erdenaki@hacettepe.edu.tr

**Keywords:** anxiety, cognitive functions, older adults, neuropsychological evaluation, geriatric psychiatry, cross-sectional study

## Abstract

**Highlights:**

**What are the main findings?**

- Older adults in the anxiety group showed selective difficulties in memory, attention, processing speed, and executive functioning despite normal global cognitive screening results.
- An anxiety-related clinical diagnosis showed a more consistent independent association with cognitive performance than anxiety symptom severity as measured by the GAS total score, alongside education, age, and depressive symptom burden.

**What are the implications of the main findings?**

- Global screening tools alone may be insufficient to identify cognitive difficulties associated with late-life anxiety, and domain-specific cognitive assessment may provide additional information.
- Clinicians should consider that cognitive difficulties associated with late-life anxiety may not be fully reflected by anxiety symptom severity alone, and domain-specific cognitive assessment may provide additional information beyond global screening tools.

**Abstract:**

Background/Objectives: Anxiety symptoms are common in older adults and are associated with cognitive changes; however, the specific pattern of deficits and the effects of anxiety diagnosis, symptom severity, and depressive symptoms remain unclear. This study compared cognitive performance between older adults with anxiety-related diagnoses and psychiatrically healthy controls and examined factors associated with cognitive outcomes. Methods: This cross-sectional study included 130 participants aged ≥ 65 years: 70 with an anxiety-related diagnosis (SCID-5-CV) and 60 controls without psychiatric diagnoses. Cognition was assessed using the SMMSE, Enhanced Cue Recall Test, Clock Drawing Test, Verbal Fluency Test, and Trail Making Test Parts A and B; anxiety and depressive symptoms were assessed with validated scales. Results: The anxiety group had higher anxiety and depression scores than controls (FDR-adjusted q < 0.001). They showed longer Trail Making Test A, B, and B–A times and lower free recall and enhanced cue recall scores (q = 0.019 and q < 0.001, respectively). No differences were observed in global cognition, clock drawing, or verbal fluency after correction. Most symptom–cognition correlations did not survive Bonferroni correction, except for state anxiety and enhanced cue recall. In regression analyses, anxiety-related diagnosis showed a more consistent association with cognition than anxiety symptom severity, along with education, age, gender, and depressive symptoms. Conclusions: Older adults with anxiety-related diagnoses showed selective impairments in processing speed, attention, executive functions, and memory despite preserved global cognitive screening. Cognitive outcomes in late-life anxiety appear more closely related to diagnostic status and other clinical/demographic factors than to anxiety severity alone.

## 1. Introduction

Together with the rapidly increasing older adult population, geriatric mental health has become a more important area of research. Anxiety disorders are one of the most common mental health problems in older adults and have been associated with negative effects on quality of life, functionality, and cognitive performance [1]. However, as anxiety symptoms in older adults can be intertwined with physical complaints, chronic diseases, or age-related changes, they can be clinically overlooked [2]. Increased anxiety is associated with an increase in psychological problems, functional loss, and the use of healthcare services, thereby showing the need for careful evaluation of this condition in the geriatric population [3,4]. Epidemiological research conducted throughout the world in general has reported that anxiety symptoms are common in older adults and prevalence estimations can significantly vary depending on the evaluation tools used [3,5].

Anxiety experienced during the ageing process is not limited to emotional problems but may also be associated with cognitive functions. In the literature, symptoms of anxiety have been reported to be associated especially with executive functions, attention, processing speed, and memory performance [6,7]. It has also been shown that anxiety and depressive symptoms can be seen together with cognitive disorder at an advanced age and mental health status may play a role in the relationship between age and cognitive deterioration [7,8,9]. Recent studies have emphasized the need to consider the clinical variables associated with mental health in addition to demographic and psychosocial variables in the evaluation of this relationship [8,10].

Therefore, the mental health symptom burden should be dealt with as an important component of cognitive performance evaluation in older adults. In this context, the concept of Mild Behavioral Impairment (MBI) is important. MBI is defined as neuropsychiatric symptoms that emerge at an older age, remain below the diagnostic threshold but disrupt functionality and last for at least 6 months. It has been suggested that these symptoms constitute a predictor for cognitive disorder and dementia. Mood disorders, including depressive symptoms and anxiety, form one of the basic categories of MBI [11]. Thus, it is thought that anxiety symptoms at an older age should be evaluated not only as an accompanying psychiatric condition but as an early sign of a potential neurodegenerative process. Most studies in the literature have been conducted in Western and East Asian populations, while comprehensive neuropsychological investigations involving older adults from non-Western settings, including Türkiye, remain scarce [5].

This study aimed to evaluate cognitive performance in older adults with clinically significant anxiety symptoms using multidimensional neuropsychological tests and to examine the effects of depressive symptom burden and demographic factors through comparison with controls without psychiatric diagnoses. The findings may contribute to a better understanding of the relationship between late-life anxiety and cognition and support more comprehensive neuropsychological evaluation in clinical practice.

## 2. Materials and Methods

This cross-sectional descriptive study included individuals aged ≥ 65 years attending the Psychiatry, Geriatrics, and Family Medicine outpatient clinics of Ankara Etlik City Hospital. Ethical approval was obtained from the Scientific Research Evaluation and Ethics Committee (AEŞH-BADEK-2024-839, 25 September 2024). All participants were literate and provided written informed consent to participate voluntarily. Between October 2024 and April 2025, 159 individuals were consecutively assessed for eligibility: the anxiety group was recruited from Psychiatry, and controls from Geriatrics or Family Medicine clinics, with stable systemic diseases.

The anxiety group comprised individuals with clinically significant anxiety symptoms who received a primary anxiety-related diagnosis (generalized anxiety disorder, unspecified anxiety disorder, or adjustment disorder with predominant anxiety) based on the SCID-5-CV; controls had no psychiatric diagnosis. Exclusion criteria for both groups were neurodevelopmental disorders, uncontrolled systemic disease interfering with cognitive assessment, and baseline SMMSE ≤ 23 (indicating possible major neurocognitive disorder). The 23/24 SMMSE cut-off was based on the validated Turkish version by Güngen et al. [12]. Major neurocognitive disorder was additionally assessed clinically by the psychiatrist during the SCID-5-CV evaluation, including functional assessment and, when available, collateral information from a relative or caregiver; participants meeting clinical criteria were excluded regardless of SMMSE score. Psychotropic medication use was not an exclusion criterion because pharmacotherapy is a primary treatment approach for anxiety disorders in older adults [2,13]. Medication use and type were systematically recorded in the anxiety group.

No formal individual matching was performed; recruitment aimed for group-level comparability in age and education, with no significant between-group differences in these variables (Table 1).

**Figure 1:**
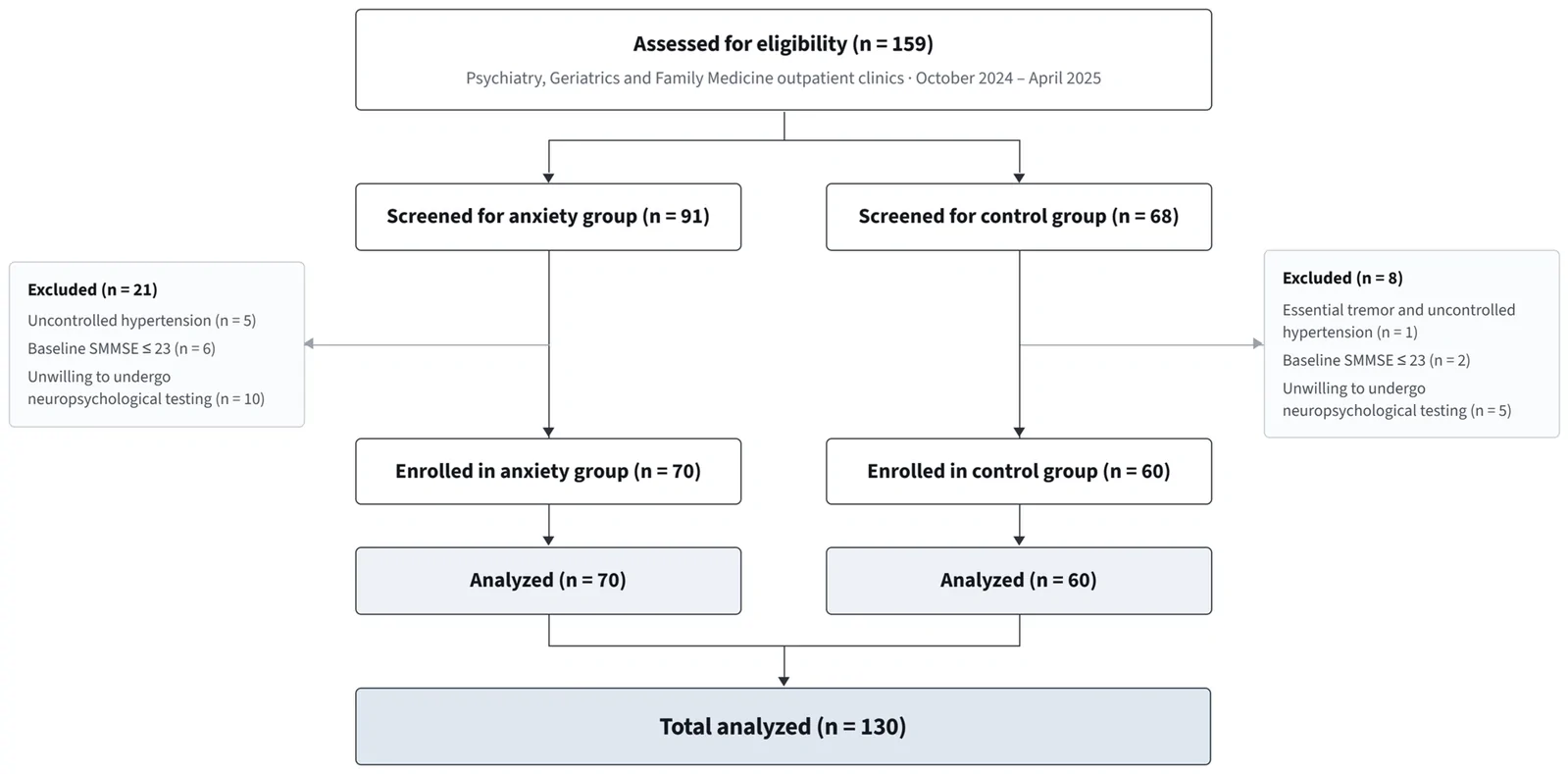
Participant flow diagram (STROBE).

Of 91 individuals screened for the anxiety group, 21 were excluded (5 for uncontrolled hypertension, 6 for SMMSE ≤ 23, and 10 for unwillingness to undergo neuropsychological testing), leaving 70 participants. Of 68 controls screened, 8 were excluded (1 for essential tremor and uncontrolled hypertension, 2 for SMMSE ≤ 23, and 5 for unwillingness to undergo testing), leaving 60 participants. Participant flow is shown in Figure 1.

An a priori power analysis using G*Power 3.1.9.7 indicated that 128 participants (64 per group) were required for the primary between-group comparisons, assuming α = 0.05, 80% power, and a medium effect size (Cohen’s d = 0.50). This calculation was not adjusted for multiple cognitive outcomes. Regression analyses were subsequently conducted as secondary exploratory analyses to examine independent associations between clinical variables and cognitive performance.

All participants were evaluated in a single session using a standardized test order. The SCID-5-CV was administered first, followed by the SMMSE, Enhanced Cue Recall Test, Clock Drawing Test, Verbal Fluency Test, and Trail Making Test Parts A and B. Anxiety and depressive symptoms were then assessed using the BAI, GAS, STAI, and GDS. The full session lasted approximately 90 min, including a fixed 15-min rest break. Most assessments were conducted by the principal investigator, a psychiatrist, and the remainder by a trained clinical psychologist. Although assessors were not blinded to diagnostic group, all cognitive tests were administered and scored using standardized protocols and objective criteria to reduce rater-dependent bias.

### 2.1. Data Collection Tools

1. Structured Clinical Interview for DSM-5 Clinician Version (SCID-5-CV): Developed by First et al. [14] and validated in Turkish by Elbir et al. [15], the SCID-5-CV was administered to all participants to establish DSM-5 diagnoses.
2. Beck Anxiety Inventory (BAI): This 21-item self-report scale assesses anxiety symptoms during the previous week [16]. Scores range from 0–63, with higher scores indicating greater anxiety [17]. The Turkish version was validated by Ulusoy [18].
3. Geriatric Anxiety Scale (GAS): This 30-item self-report scale assesses anxiety severity in older adults [19] and was validated in Turkish by Karahan [20]. It includes somatic, cognitive, and affective subscales, with items scored 0–3; higher scores indicate greater anxiety.
4. State-Trait Anxiety Inventory (STAI): Developed by Spielberger et al. [21] and validated in Turkish by Le Compte and Öner [22], the STAI assesses state and trait anxiety. Each 20-item form yields a score of 20–80, with higher scores indicating greater anxiety.
5. Geriatric Depression Scale (GDS): This 30-item self-report scale assesses depressive symptoms in older adults [23] and was validated in Turkish by Ertan and Eker [24]. Scores range from 0–30, with higher scores indicating greater depressive symptoms.
6. Standardized Mini-Mental State Examination (SMMSE): This 30-point screening test assesses global cognition, including orientation, registration, attention/calculation, recall, and language. The Turkish version was validated by Güngen et al. [12].
7. Enhanced Cue Recall Test: This test assesses memory processes and distinguishes registration/storage from recall. The Turkish version was validated by Saka et al. [25]. The maximum score is 48, with scores < 41 indicating memory impairment.
8. Clock Drawing Test: This brief cognitive screening test assesses visuoconstructional skills, planning, and executive functions. Its Turkish validity and reliability were established by Cangöz et al. [26].
9. Verbal Fluency Test: This test requires producing words within a specified time according to a category or initial letter [27]. Phonemic and categorical fluency provide information on language access, semantic memory, and executive functions [28].
10. Trail Making Test: Parts A and B assess attention, processing speed, visual scanning, cognitive flexibility, and executive functions. The Turkish standardization study was conducted by Cangöz et al. [29]. No fixed maximum completion time was prespecified; completion time was recorded until task completion, and all participants completed Parts A and B.

### 2.2. Statistical Analysis

Data were analyzed using IBM SPSS Statistics, version 31.0 (IBM Corp., Armonk, NY, USA). Continuous variables were summarized as mean ± SD, median, minimum, and maximum, and categorical variables as *n* (%). No data were missing, so analyses used the full sample (*n* = 130) without imputation. No single cognitive measure or formal set of coprimary outcomes was prespecified. Between-group comparisons across the cognitive battery constituted the main analyses, while correlation, regression, and MANOVA analyses were considered exploratory secondary analyses.

Normality was assessed using the Shapiro–Wilk test [30], and homogeneity of variances using Levene’s test. Between-group comparisons used independent-samples *t*-tests for normally distributed variables, Mann–Whitney U-tests for non-normal variables, and Welch’s *t*-test when variance homogeneity was violated. For between-group differences, Hodges–Lehmann estimates with bootstrap 95% CIs were reported for Mann–Whitney U tests, while 95% CIs for mean differences were reported for *t*-tests. Categorical variables were presented in contingency tables and analyzed using Pearson’s chi-square or exact tests, as appropriate. For multiple comparisons, between-group *p*-values were adjusted using the Benjamini–Hochberg false discovery rate (FDR) procedure. Associations between continuous variables were examined using Pearson’s or Spearman’s correlations, as appropriate. Correlation and regression analyses were exploratory secondary analyses. Bonferroni correction was applied to 88 correlation tests, yielding an adjusted significance threshold of α = 0.000568.

As additional exploratory analyses, MANOVAs assessed overall group effects on psychological symptoms and cognitive performance. The psychological MANOVA included BAI, GDS, STAI-1, STAI-2, and GAS total scores, while the cognitive MANOVA included SMMSE, Clock Drawing, Verbal Fluency, Trail Making Test A and B times, and Enhanced Cue Recall scores. Because homogeneity assumptions were violated, Pillai’s Trace was used as the primary statistic. Multivariate outliers were assessed separately within each group using Mahalanobis distance with a chi-square criterion of *p* < 0.001.

Multiple linear regression analyses using the Enter method were performed to identify independent factors associated with cognitive outcomes. Age, gender, education, and depressive symptom burden (GDS total score) were included as clinically relevant covariates. Whole-sample models included diagnostic group as the main predictor together with age, gender, education, and GDS score. Anxiety-group models included GAS total score and disease duration, along with age, gender, education, and GDS score, to examine the associations of anxiety severity and illness chronicity with cognition.

Regression assumptions were assessed using Durbin–Watson statistics (1.5–2.5), tolerance (>0.200), VIF (<3), residual normality, predicted-versus-residual plots for homoscedasticity, and Cook’s distance (<1). Unless otherwise specified, *p* < 0.05 was considered statistically significant.

Effect sizes were calculated for significant between-group differences. Cohen’s d was used for independent-samples *t*-tests (0.20 small, 0.50 moderate, ≥0.80 large), and r for Mann–Whitney U-tests (0.10 small, 0.30 moderate, ≥0.50 large) [31,32].

## 3. Results

A total of 130 participants were included: 70 (53.8%) in the anxiety group and 60 (46.2%) controls. The groups did not differ significantly in age, gender, education, marital or employment status, living arrangements, smoking, or alcohol use (*p* > 0.05) (Table 1). Chronic medical conditions were present in 62/70 (88.6%) participants in the anxiety group and 53/60 (88.3%) controls, with no between-group difference (χ^2^ = 0.00, *p* = 1.000). In the anxiety group, mean disease duration was 9.34 ± 7.88 years; primary diagnoses were generalized anxiety disorder in 32 (45.7%), unspecified anxiety disorder in 26 (37.1%), and adjustment disorder with predominant anxiety in 12 (17.1%). Seventeen participants (24.3%) had psychiatric comorbidity, including major depressive disorder in 11 (15.7%), adjustment disorder in 3 (4.3%), panic disorder in 2 (2.9%), and a specific phobia in 1 (1.4%). Antidepressants were used by 65 participants (92.9%), while 5 (7.1%) used no psychotropic medication. Three (4.3%) used benzodiazepines as needed but not on the test day, and 1 (1.4%) received adjunctive antipsychotic treatment. No participant used a separate sedative/hypnotic medication. Four medicated participants (6.2%) used more than two psychotropic drugs concurrently.

Anxiety and depression scores were significantly higher in the anxiety group than in controls (FDR-adjusted q < 0.001 for all) (Table 2). Large effect sizes were observed for BAI, GDS, STAI-1, STAI-2, GAS Cognitive, and GAS total scores, and moderate effects for GAS Somatic and Mood subscales. Exploratory MANOVA also showed a significant overall group effect across BAI, GDS, STAI-1, STAI-2, and GAS total scores (Pillai’s Trace = 0.418, F(5, 124) = 17.822, *p* < 0.001, partial η^2^ = 0.418) (Table S1).

The differences between the Anxiety and Control groups in respect of the cognitive function variables are presented in Table 3. When evaluated in terms of cognitive functions, the Trail Making A and B times and the B–A difference were determined to be at a significantly higher level in the anxiety group than in the control group (FDR-adjusted q < 0.001). As longer times in the Trail Making test reflect a lower performance, this finding indicates poorer performance in attention, processing speed, and executive functions in the Anxiety group. The free recall total points (FDR-adjusted q = 0.019) and enhanced cue recall points (FDR-adjusted q < 0.001) were found to be significantly lower in the anxiety group, indicating poorer memory performance. An exploratory MANOVA was conducted to examine the overall difference in cognitive performance between the Anxiety and Control groups. Because the assumption of homogeneity of covariance matrices was violated (Box’s M = 133.386, F = 6.031, *p* < 0.001), Pillai’s Trace was used as the primary multivariate statistic. A statistically significant overall multivariate group effect was observed (Pillai’s Trace = 0.257, F(6, 123) = 7.094, *p* < 0.001, partial η^2^ = 0.257) (Table S2).

No significant between-group differences were found in SMMSE, Clock Drawing, or Verbal Fluency scores after FDR correction (q > 0.05) (Table 3). Using the validated Enhanced Cue Recall cut-off of <41, 10/70 (14.3%) participants in the anxiety group and 4/60 (6.7%) controls scored below the threshold; this difference was not statistically significant (Fisher’s exact *p* = 0.256; OR = 2.33). In the anxiety group, although several correlations between cognitive scores and anxiety/depression measures were nominally significant, none remained significant after Bonferroni correction for 88 comparisons (α = 0.000568) (Figure S1A).

In the control group, relationships between cognitive test scores and anxiety/depression scale scores were examined using Pearson or Spearman correlations, as appropriate. A negative correlation between Enhanced Cue Recall and STAI-1 state anxiety was observed at the unadjusted level (r_s_ = −0.363, raw *p* = 0.004), but did not remain significant after Bonferroni correction for 88 comparisons (adjusted *p* = 0.352; α = 0.000568). No other correlations remained significant after correction (Figure S1B).

Whole-sample correlations are presented in Table 4. After Bonferroni correction for 88 comparisons (α = 0.000568), only the negative correlation between Enhanced Cue Recall and STAI-1 state anxiety remained significant (r_s_ = −0.333, raw *p* = 0.000108, adjusted *p* = 0.010), indicating that higher state anxiety was associated with lower Enhanced Cue Recall scores. Although several other correlations were nominally significant before correction, none remained significant after correction.

Regression results for the whole sample and anxiety group are summarized in Table 5. All whole-sample models were significant. For Trail Making Test Part A, group was a positive predictor (β = 0.298; *p* < 0.05) and education a negative predictor (β = −0.414; *p* < 0.05), with the model explaining 28.7% of the variance. For Parts B and B–A, group (β = 0.490 and 0.504) and age (β = 0.178 for both) were positive predictors, while education was a negative predictor (β = −0.337 and −0.268; all *p* < 0.05); depression was also a negative predictor of B–A (β = −0.209; *p* < 0.05). In Verbal Fluency models, age was a negative predictor (β = −0.271 and −0.267) and education a positive predictor (β = 0.397 and 0.490; *p* < 0.05), while depression negatively predicted categorical fluency (β = −0.202; *p* < 0.05). For Enhanced Cue Recall, group (β = −0.348) and age (β = −0.275) were negative predictors (*p* < 0.05). Diagnostic values were within acceptable ranges: Durbin–Watson 1.632–2.067, tolerance ≥ 0.652, VIF ≤ 1.534, and Cook’s distance ≤ 0.560.

Within the anxiety group, education and age were significant predictors across most cognitive domains. Male gender significantly predicted Trail Making Test Part B, B–A difference, and Verbal Fluency performance. The Enhanced Cue Recall model was not significant, and GAS total score was not an independent predictor in any model (gender was a nominal predictor of Enhanced Cue Recall, though the overall model was not significant). Model diagnostics were acceptable: Durbin–Watson 1.875–2.171, tolerance ≥ 0.452, VIF ≤ 2.210, and Cook’s distance ≤ 0.494 (Table 5).

## 4. Discussion

In this study, cognitive functions and psychological symptoms of older adults with anxiety-related clinical diagnoses were compared with those of individuals without psychiatric diagnoses. Participants in the anxiety group had higher anxiety and depressive symptoms and poorer performance in processing speed, attention, executive functions, and memory, despite preserved global cognitive screening performance. These findings suggest that late-life anxiety may be associated with selective cognitive difficulties not detected by brief screening instruments. Given the cross-sectional design, these findings reflect associations rather than causal relationships between anxiety and cognitive performance.

The anxiety group included a higher proportion of women, consistent with previous studies reporting a higher prevalence of anxiety disorders among older women [5]. The anxiety and control groups were comparable in key demographic characteristics, particularly age and education, although residual confounding should be considered when interpreting cognitive findings. The mean disease duration suggested a prolonged clinical course for many participants. The anxiety group included different anxiety-related diagnoses and psychiatric comorbidities, reflecting the clinical heterogeneity commonly observed in late-life anxiety. Therefore, the findings should be interpreted in the context of overall psychiatric symptom burden and clinical heterogeneity rather than as a cognitive profile specific to a single anxiety disorder.

Higher anxiety and depressive symptom levels in the anxiety group supported clinical differentiation between the groups and indicated that depressive symptoms should be considered when interpreting cognitive findings. Anxiety measures demonstrated consistent between-group differences across the applied instruments. However, these measures assess partially overlapping but distinct dimensions of anxiety, and direct comparison or ranking of effect sizes across instruments was not considered appropriate. Given the heterogeneity of anxiety presentations in older adults, the selection of appropriate assessment tools with adequate age-related validity is important for accurate clinical evaluation [33].

Longer Trail Making Test A and B times and a greater B–A difference in the anxiety group indicated poorer processing speed, attention, and executive functioning, while lower free and enhanced cue recall scores indicated poorer memory performance. These findings are consistent with models suggesting that reduced attentional control in anxiety may impair information processing and memory retrieval [34]. In contrast, similar SMMSE, Clock Drawing, and Verbal Fluency scores between groups suggest selective rather than generalized cognitive difficulties and highlight the limitations of brief global screening tools in detecting domain-specific deficits. Overall, these findings are consistent with previous studies linking late-life anxiety to poorer processing speed, attention, executive functioning, and memory [6,7,8,35].

Within the anxiety group, no correlations between anxiety or depressive symptoms and cognitive scores remained significant after Bonferroni correction; similarly, the state anxiety–enhanced cue recall correlation in controls did not remain significant. These findings do not support a consistent dose–response relationship between symptom severity and cognitive performance, suggesting anxiety symptom severity alone may therefore not fully explain the cognitive differences between groups, and that broader clinical and demographic factors may contribute [6,8,35].

In the whole sample, several associations between anxiety/depressive symptoms and cognitive performance were nominally significant, but after correction only the negative association between state anxiety and enhanced cue recall remained significant. Because the analysis combined both groups, this association may partly reflect between-group differences and other clinical factors, including depressive symptom burden. Attention Control Theory suggests that anxiety may impair attentional control during cognitive tasks, contributing to executive and memory difficulties [6,34]. Overall, the limited robust associations support a multifactorial and heterogeneous relationship between late-life anxiety, depression, and cognition [6,7,8,10,35,36], indicating that cognitive effects should be interpreted alongside diagnostic status and other demographic and clinical factors.

Depression has been associated with increased dementia risk, although causality remains uncertain. Anxiety has also been proposed as a potential contributor to cognitive decline or as a manifestation of early neurobiological changes [9,37,38,39]. The Mild Behavioral Impairment (MBI) framework provides a theoretical perspective through which associations between anxiety symptoms and cognitive vulnerability may be further explored. However, MBI was not directly assessed in this study, and the mean illness duration of approximately nine years indicates a predominantly long-standing clinical course. Therefore, the present findings should not be interpreted as evidence that anxiety represents an early or prodromal neurodegenerative manifestation in this sample. A meta-analysis of prospective cohorts similarly associated anxiety with increased dementia risk, although causality remains unresolved [11]. While our findings do not establish a temporal or causal relationship, they support considering anxiety within broader neuropsychiatric risk frameworks in older adults. Longitudinal studies with repeated cognitive assessments are needed to clarify the temporal and potentially causal nature of this relationship.

The high rate of antidepressant use in the anxiety group is consistent with clinical practice, where pharmacotherapy is commonly used for anxiety disorders in older adults [2,13]. Antidepressants may have variable, neutral, or modest cognitive effects in older adults [40]. However, the lack of an adequately sized unmedicated subgroup prevents separating medication effects from those associated with anxiety-related clinical diagnosis and related characteristics. Therefore, the independent contributions of diagnosis, symptom burden, and psychotropic medication use to the observed cognitive differences cannot be determined.

In whole-sample regression models, diagnostic group was independently associated with Trail Making Test A, B, and B–A times and Enhanced Cue Recall performance. However, this association should not be interpreted as a specific effect of anxiety per se: the clinical group also differed from controls in antidepressant use, depressive comorbidity, and other clinical characteristics that could not be fully disentangled from diagnostic status in this naturalistic sample. In contrast, GAS total score was not independently associated with cognition within the anxiety group after adjustment for demographic and clinical variables. These findings suggest that anxiety-related diagnostic status may capture cognitive differences beyond symptom severity alone, potentially reflecting the broader clinical profile associated with this status rather than anxiety symptoms in isolation, consistent with previous studies [6,8,41]. Associations across processing speed, executive function, and memory also argue against a purely domain-specific finding, although these exploratory results require confirmation.

Education was consistently associated with cognitive performance across several domains, while age was associated with executive function, verbal fluency, and recall. Depressive symptom burden remained associated with selected outcomes, including Trail Making Test B–A difference and categorical verbal fluency, and male gender was associated with several cognitive measures within the anxiety group [7,8]. Overall, these findings suggest that cognitive performance in late-life anxiety reflects a combination of diagnostic status, education, age, gender, and depressive symptom burden rather than anxiety symptom severity alone.

Several strengths should be highlighted. First, this study provides a comprehensive assessment of cognitive functioning in older adults with clinically significant anxiety symptoms, including attention, processing speed, executive functions, and memory. Second, the groups were comparable in key demographic characteristics, particularly age and education, and potential demographic and clinical effects were examined through adjusted analyses. Third, cognitive assessment extended beyond global screening to multiple neuropsychological domains. Another strength was the separate evaluation of anxiety-related clinical diagnosis and symptom severity, allowing their associations with cognition to be examined independently. Finally, psychiatric diagnoses were established using SCID-5, and anxiety was assessed with multiple psychometric instruments covering different symptom dimensions.

### Limitations

This study has several limitations. First, its cross-sectional design precludes causal interpretations of the relationship between anxiety and cognitive functioning. Second, the single-centre design and relatively modest sample size may limit generalizability. The clinical heterogeneity of the anxiety group restricted evaluation of disorder-specific cognitive profiles, and potentially relevant factors such as medical comorbidity severity, sleep quality, and social support could not be assessed in detail. Mild cognitive impairment (MCI) was not systematically assessed with a dedicated validated instrument or diagnostic procedure and therefore could not be excluded as a separate diagnostic category; consequently, some participants with relatively low cognitive scores may have had an MCI-range profile. Although psychotropic medication use was systematically recorded, most participants in the anxiety group were receiving antidepressants (92.9%, *n* = 65), with only five medication-free, preventing full separation of pharmacotherapy effects from those associated with anxiety-related clinical diagnosis and related clinical characteristics. Detailed data on antidepressant subclasses and dosages were also unavailable. Because antidepressant use was closely linked to diagnostic status in this naturalistic sample, their independent contributions could not be reliably disentangled. Assessors were not blinded to diagnostic group, although standardized instruments with criterion-based scoring may have reduced this bias. Despite a standardized assessment sequence and scheduled rest period, order and fatigue effects cannot be fully excluded. As STAI-State was administered after neuropsychological testing, it may not fully reflect anxiety levels during cognitive assessment, which should be considered when interpreting state–anxiety correlations. FDR correction was applied to group comparisons and Bonferroni correction to correlation analyses. The anxiety-group regression models (*n* = 70, six predictors; approximately 12 cases per predictor) may have been comparatively underpowered; accordingly, these exploratory findings require confirmation in larger samples.

## 5. Conclusions

The findings of this study demonstrated that, compared with controls without a psychiatric diagnosis, older adults in the anxiety group had higher anxiety and depressive symptom levels and showed poorer performance in selected cognitive domains, particularly memory, processing speed, and executive functions. Regression analyses indicated that the presence of an anxiety-related clinical diagnosis showed a more consistent association with cognitive performance than anxiety symptom severity as measured by the GAS total score, alongside other relevant factors including education, age, gender, and depressive symptom burden. These findings suggest that cognitive performance in older adults with anxiety should be evaluated in the context of diagnostic status and broader demographic and clinical factors rather than anxiety symptom severity alone. Future longitudinal studies with repeated cognitive assessments are needed to clarify the temporal relationship between anxiety symptoms and cognitive functioning.

## Figures and Tables

**Table 1:** Sociodemographic and clinical characteristics of the study participants.

Variable	Anxiety Group *n* = 70	Control Group *n* = 60	Test Statistic	*p*-Value
Age (years), mean ± SD; median (min–max)	70.30 ± 4.35; 70.00 (65–86)	70.10 ± 5.02; 69.00 (65–84)	U = 1937.00	0.444
Years of education, mean ± SD; median (min–max)	7.13 ± 3.38; 5.00 (2–15)	8.32 ± 4.05; 7.00 (2–17)	U = 1767.00	0.098
Gender, n (%)				
Female	52 (74.3)	36 (60.0)	χ^2^ = 2.397	0.122
Male	18 (25.7)	24 (40.0)		
Marital status, n (%)				
Married	46 (65.7)	44 (73.3)	χ^2^ = 1.370	0.713
Single	4 (5.7)	4 (6.7)		
Widowed	14 (20.0)	9 (15.0)		
Divorced	6 (8.6)	3 (5.0)		
Employment status, n (%)				
Never worked	38 (54.3)	22 (36.7)	χ^2^ = 4.084	0.130
Retired	29 (41.4)	35 (58.3)		
Still working	3 (4.3)	3 (5.0)		
Living arrangements, n (%)				
Living alone	13 (18.6)	9 (15.0)	χ^2^ = 0.351	0.839
Living with spouse	49 (70.0)	43 (71.7)		
Living with relatives	8 (11.4)	8 (13.3)		
Alcohol use, n (%)				
No	70 (100.0)	59 (98.3)	χ^2^ = 1.176	0.462
Yes	0 (0.0)	1 (1.7)		
Smoking status, n (%)				
Non-smoker	65 (92.9)	56 (93.3)	χ^2^ = 0.011	1.000
Smoker	5 (7.1)	4 (6.7)		
Clinical Characteristics of The Anxiety Group				
Variable			Mean ± SD; Median (min–max)	
Disease duration (years)			9.34 ± 7.88; 10 (2–50)	
Primary psychiatric diagnosis			n	(%) *
Generalized anxiety disorder			32	45.7
Unspecified anxiety disorder			26	37.1
Adjustment disorder with anxiety as the predominant clinical feature			12	17.1
Additional psychiatric comorbidity				
No additional psychiatric diagnosis			53	75.7
≥1 additional psychiatric diagnosis			17	24.3
Comorbid psychiatric diagnosis				
Major depressive disorder			11	15.7
Adjustment disorder			3	4.3
Panic disorder			2	2.9
Specific phobia			1	1.4

SD, standard deviation; χ^2^, chi-square test. U, Mann–Whitney U test. * Percentages for primary psychiatric diagnoses and psychiatric comorbidities were calculated using the anxiety-group sample (*n* = 70) as the denominator. Primary diagnoses are mutually exclusive and sum to 100%; comorbid diagnoses are reported separately and may overlap with the primary diagnoses.

**Table 2:** Results related to the anxiety variables in the Anxiety and Control groups.

Variable	Group	Mean ± SD	Median (Min–Max)	Test Statistic	*p*	FDR q	Effect Size	95% CI
Beck Anxiety Inventory Total Points	Anxiety	18.84 ± 10.733	17 (2–48)	U = 589.50	<0.001	<0.001	r = 0.619	8.00–13.00
	Control	7.05 ± 5.694	6 (0–28)					
Geriatric Depression Scale Total Points	Anxiety	13.36 ± 6.494	14 (2–28)	U = 692.50	<0.001	<0.001	r = 0.577	6.00–10.00
	Control	5.57 ± 4.168	5 (0–16)					
STAI-1 State Anxiety Total Points	Anxiety	40.91 ± 9.650	41 (22–66)	U = 860.50	<0.001	<0.001	r = 0.508	8.00–14.00
	Control	30.72 ± 7.891	30.5 (20–50)					
STAI-2 Trait Anxiety Total Points	Anxiety	47.50 ± 8.364	47 (29–65)	t = 7.868 ^1^	<0.001	<0.001	d = 1.353	7.53–12.60
	Control	37.43 ± 6.185	38 (24–51)					
GAS–Somatic (physical) Subscale	Anxiety	8.66 ± 4.742	8 (0–18)	U = 942.00	<0.001	<0.001	r = 0.476	3.00–6.00
	Control	4.30 ± 3.027	4 (0–14)					
GAS–Cognitive Subscale	Anxiety	6.80 ± 5.174	5 (0–21)	U = 870.00	<0.001	<0.001	r = 0.508	2.00–5.00
	Control	2.30 ± 2.884	1 (0–12)					
GAS–Mood Subscale	Anxiety	6.81 ± 4.617	6 (0–18)	U = 964.00	<0.001	<0.001	r = 0.467	2.00–5.00
	Control	2.78 ± 2.669	2 (0–11)					
Geriatric Anxiety Scale Total Points	Anxiety	22.27 ± 12.931	19.5 (0–52)	U = 824.00	<0.001	<0.001	r = 0.523	8.00–16.00
	Control	9.38 ± 7.818	7.5 (0–32)					

SD, standard deviation; U, Mann–Whitney U test; t, independent-samples *t*-test; ^1^, Welch’s *t*-test; FDR, false discovery rate; r, effect size for the Mann–Whitney U test; d, Cohen’s d; CI, confidence interval for the difference between the Anxiety and Control groups; GAS, Geriatric Anxiety Scale.

**Table 3:** Results related to the cognitive function variables in the Anxiety and Control groups.

Variable	Group	Mean ± SD	Median (Min–Max)	Test Statistic	*p*	FDR q	Effect Size	95% CI
SMMSE Total Points	Anxiety	27.04 ± 1.740	27 (23–30)	U = 1978.50	0.564	0.564	-	−1.00–0.00
	Control	27.23 ± 1.741	27 (23–30)					
Clock Drawing Total Points	Anxiety	3.51 ± 0.717	4 (1–4)	U = 1965.00	0.455	0.501	-	0.00–0.00
	Control	3.63 ± 0.551	4 (2–4)					
Verbal Fluency Phonemic Points	Anxiety	18.07 ± 9.825	16 (3–47)	U = 1779.00	0.133	0.183	-	−6.00–1.00
	Control	21.07 ± 11.240	18.5 (6–50)					
Verbal Fluency Categorical Points	Anxiety	49.61 ± 13.411	48 (27–93)	U = 1638.50	0.031	0.057	-	−9.00–0.00
	Control	53.42 ± 11.854	53.5 (28–85)					
Verbal Fluency Total Test Points	Anxiety	67.69 ± 20.998	65 (34–127)	U = 1664.50	0.042	0.066	-	−14.00–0.00
	Control	74.48 ± 21.162	71 (34–135)					
Trail Making Test–Part A Time	Anxiety	76.76 ± 36.471	67 (26–240)	U = 1073.50	<0.001	<0.001	r = 0.421	12.99–28.00
	Control	52.42 ± 20.061	47.5 (26–127)					
Trail Making Test–Part B Time	Anxiety	199.24 ± 125.639	168 (46–560)	U = 856.00	<0.001	<0.001	r = 0.510	48.99–97.00
	Control	102.98 ± 45.513	101.5 (48–355)					
Trail Making Test B–A Difference	Anxiety	122.49 ± 102.636	94 (13–473)	U = 915.00	<0.001	<0.001	r = 0.485	31.00–65.01
	Control	50.57 ± 33.154	46.5 (10–228)					
Enhanced Cue Recall Points	Anxiety	43.67 ± 4.806	45 (22–48)	U = 1017.00	<0.001	<0.001	r = 0.460	−3.00–−1.00
	Control	46.75 ± 2.447	48 (38–48)					
Free Recall Total Points	Anxiety	28.79 ± 5.626	29 (11–40)	t = −2.668	0.009	0.019	d = 0.469	−4.29–−0.64
	Control	31.25 ± 4.775	32 (19–41)					
Cue Recall Total Points	Anxiety	14.89 ± 3.512	15 (6–24)	t = −1.001	0.319	0.390	-	−1.83–0.60
	Control	15.50 ± 3.457	15 (7–23)					

SD, standard deviation; U, Mann–Whitney U test; t, independent-samples *t*-test; FDR, false discovery rate; r, effect size for the Mann–Whitney U test; d, Cohen’s d; CI, confidence interval for the difference between the Anxiety and Control groups.

**Table 4:** Correlations associated with the anxiety and cognitive function variables in the whole sample.

		BAI Total Points ^1^	Geriatric Depression Scale Total Points ^1^	STAI-1 State Anxiety Total Points ^1^	STAI-2 Trait Anxiety Total Points	Somatic (Physical) Subscale ^1^	Cognitive Subscale ^1^	Mood Subscale ^1^	Geriatric Anxiety Scale Total Points ^1^
SMMSE Total Points ^1^	r	−0.038	−0.151	−0.125	−0.109	0.006	−0.028	−0.032	−0.020
	*p*	0.668	0.086	0.156	0.217	0.943	0.751	0.718	0.820
Clock Drawing Total Points ^1^	r	0.127	−0.001	−0.020	0.110	0.108	0.042	0.098	0.088
	*p*	0.150	0.988	0.824	0.215	0.222	0.634	0.266	0.320
Verbal Fluency Phonemic Points ^1^	r	−0.096	−0.089	−0.150	−0.120	−0.037	−0.044	−0.073	−0.055
	*p*	0.275	0.312	0.089	0.174	0.677	0.620	0.412	0.533
Verbal Fluency Categorical Points ^1^	r	−0.260	−0.279	−0.219	−0.237	−0.169	−0.200	−0.189	−0.204
	*p*	0.003	0.001	0.012	0.007	0.054	0.022	0.031	0.020
Verbal Fluency Test Points ^1^	r	−0.202	−0.203	−0.201	−0.206	−0.121	−0.144	−0.151	−0.152
	*p*	0.021	0.020	0.022	0.019	0.171	0.101	0.086	0.084
Trail Making Test–Part A Time ^1^	r	0.249	0.271	0.205	0.225	0.123	0.131	0.126	0.141
	*p*	0.004	0.002	0.019	0.010	0.165	0.137	0.154	0.109
Trail Making Test–Part B Time ^1^	r	0.282	0.268	0.203	0.225	0.161	0.174	0.194	0.200
	*p*	0.001	0.002	0.020	0.010	0.067	0.048	0.027	0.022
Trail Making Test B–A Difference ^1^	r	0.242	0.203	0.162	0.186	0.148	0.150	0.180	0.185
	*p*	0.006	0.021	0.065	0.035	0.093	0.089	0.041	0.035
Free Recall Total Points ^1^	r	−0.112	−0.141	−0.206	−0.161	−0.034	−0.027	−0.079	−0.054
	*p*	0.203	0.110	0.019	0.067	0.698	0.758	0.372	0.538
Cue Recall Total Points	r	−0.063	−0.037	0.032	−0.055	−0.088	−0.090	−0.111	−0.110
	*p*	0.480	0.674	0.721	0.538	0.317	0.307	0.207	0.213
Enhanced Cue Recall Points ^1^	r	−0.234	−0.271	−0.333 *	−0.279	−0.161	−0.145	−0.242	−0.210
	*p*	0.007	0.002	<0.001	0.001	0.067	0.100	0.005	0.016

^1^ Spearman’s correlation. Bonferroni correction was applied across 88 correlation tests (α = 0.000568). * Significant after Bonferroni correction.

**Table 5:** Results of the regression analyses of the cognitive function variables of the whole sample and of the anxiety group.

Dependent Variable		Group β/B [95% CI]	Age β/B [95% CI]	Years of Education β/B [95% CI]	Gender β/B [95% CI]	GDS β/B [95% CI]	Adj. R^2^	F
Whole Sample								
Trail Making Test—Part A Time		0.30/19.24 * [7.47, 31.01]	0.12/0.85 [−0.20, 1.91]	−0.41/−3.58 * [−4.91, −2.25]	0.01/0.99 [−9.91, 11.90]	0.02/0.11 [−0.76, 0.97]	0.29	11.38 *
Trail Making Test—Part B Time		0.49/106.03 * [67.58, 144.49]	0.18/4.15 * [0.69, 7.60]	−0.34/−9.78 * [−14.12, −5.43]	0.07/15.87 [−19.77, 51.50]	−0.16/−2.56 [−5.38, 0.26]	0.32	13.21 *
Trail Making Test B—A Difference		0.50/86.79 * [55.07, 118.51]	0.18/3.30 * [0.45, 6.15]	−0.27/−6.20 * [−9.78, −2.61]	0.08/14.87 [−14.52, 44.27]	−0.21/−2.67 * [−4.99, −0.34]	0.27	10.67 *
Verbal Fluency Categorical Points		0.02/0.48 [−4.35, 5.30]	−0.27/−0.75 * [−1.18, −0.31]	0.40/1.36 * [0.81, 1.91]	−0.12/−3.32 [−7.79, 1.16]	−0.20/−0.38 * [−0.74, −0.03]	0.24	9.02 *
Verbal Fluency Test Points		−0.01/−0.26 [−7.95, 7.43]	−0.27/−1.22 * [−1.91, −0.53]	0.49/2.79 * [1.92, 3.66]	−0.10/−4.59 [−11.71, 2.54]	−0.15/−0.47 [−1.03, 0.10]	0.30	11.90 *
Enhanced Cue Recall Points		−0.35/−2.91 * [−4.52, −1.29]	−0.28/−0.25 * [−0.39, −0.10]	0.16/0.18 [−0.01, 0.36]	−0.02/−0.21 [−1.71, 1.28]	0.01/0.01 [−0.11, 0.13]	0.20	7.42 *
Anxiety Group								
Dependent variable	Age β/B [95% CI (B)]	Years of education β/B [95% CI (B)]	Gender β/B [95% CI (B)]	GDS β/B [95% CI (B)]	GAS β/B [95% CI (B)]	Disease duration β/B [95% CI (B)]	Adj. R^2^	F
Trail Making Test—Part A Time	0.10/0.85 [−1.10, 2.80]	−0.57/−5.99 * [−8.72, −3.26]	0.18/14.38 [−5.96, 34.71]	0.19/1.03 [−0.80, 2.85]	−0.17/−0.47 [−1.36, 0.43]	0.05/0.25 [−0.83, 1.33]	0.20	3.69 *
Trail Making Test—Part B Time	0.27/7.14 * [1.27, 13.00]	−0.53/−17.83 * [−26.04, −9.62]	0.28/74.30 * [13.11, 135.49]	0.01/0.24 [−5.26, 5.73]	−0.17/−1.49 [−4.18, 1.20]	−0.14/−2.03 [−5.28, 1.22]	0.30	5.48 *
Trail Making Test B—A Difference	0.29/6.29 * [1.40, 11.18]	−0.43/−11.84 * [−18.69, −4.99]	0.28/59.92 * [8.90, 110.94]	−0.06/−0.79 [−5.37, 3.79]	−0.14/−1.03 [−3.27, 1.22]	−0.19/−2.28 [−4.99, 0.43]	0.27	4.85 *
Verbal Fluency—Categorical Points	−0.37/−1.16 * [−1.87, −0.45]	0.35/1.40 * [0.40, 2.39]	−0.31/−9.62 * [−17.04, −2.21]	−0.16/−0.33 [−1.00, 0.33]	−0.05/−0.05 [−0.38, 0.28]	0.05/0.08 [−0.31, 0.48]	0.26	4.77 *
Verbal Fluency Test Points	−0.36/−1.77 * [−2.84, −0.69]	0.47/2.94 * [1.43, 4.45]	−0.26/−12.30 * [−23.52, −1.08]	−0.13/−0.42 [−1.28, 0.59]	−0.04/−0.07 [−0.56, 0.43]	0.07/0.18 [−0.42, 0.78]	0.30	5.52 *
Enhanced Cued Recall Points	−0.22/−0.22 [−0.46, 0.02]	0.20/0.23 [−0.09, 0.55]	−0.29/−2.55 * [−4.93, −0.18]	−0.08/−0.05 [−0.26, 0.16]	0.18/0.05 [−0.05, 0.16]	−0.02/−0.01 [−0.17, 0.15]	0.09	1.99

* *p* < 0.05 Diagnostic group was coded as 0 = Control and 1 = Anxiety group; gender was coded as 0 = Female and 1 = Male. GAS: Geriatric Anxiety Scale, GDS: Geriatric Depression Scale.

## Data Availability

The datasets generated and/or analysed during the current study are not publicly available due personal details but are available from the corresponding author on reasonable request.

## Supplementary Materials

The following supporting information can be downloaded at: https://www.mdpi.com/article/10.3390/brainsci16090949/s1, Figure S1A: Heatmap for the Anxiety Group (n = 70); Figure S1B: Heatmap for the Control Group (n = 60); Table S1: Exploratory MANOVA and Assumption Checks for the Comparison of the Anxiety and Control Groups (Table 2); Table S2: Exploratory MANOVA and Assumption Checks for Cognitive Performance in the Anxiety and Control Groups (Table 3).

## Abbreviations

The following abbreviations are used in this manuscript:

BAI	Beck Anxiety Inventory
STAI	State-Trait Anxiety Inventory
STAI-1	State-Trait Anxiety Inventory, State Anxiety Form
STAI-2	State-Trait Anxiety Inventory, Trait Anxiety Form
SCID-5	Structured Clinical Interview for DSM-5
SCID-5-CV	Structured Clinical Interview for DSM-5, Clinician Version
aOR	Adjusted odds ratio
CI	Confidence interval
SMMSE	Standardized Mini-Mental State Examination
GAS	Geriatric Anxiety Scale
GDS	Geriatric Depression Scale
DSM-5	Diagnostic and Statistical Manual of Mental Disorders, Fifth Edition
SPSS	Statistical Package for the Social Sciences
SD	Standard deviation
ANOVA	Analysis of variance
MBI	Mild Behavioral Impairment
OR	Odds ratio
SE	Standard error
