# Supplementary material for: Neuropsychological Correlates of Anxiety in Older Adults: A Cross-Sectional Comparison with Controls Without a Psychiatric Diagnosis
Source: Brain Sci. 2026 Sep 5;16(9):949. doi: 10.3390/brainsci16090949 (PMC13604897; doi:10.3390/brainsci16090949)
Supplement: Supplementary file 1 [file brainsci-16-00949-s001.zip › brainsci-4508954-supplementary-supp-revised.pdf]

Supplementary Analysis – Exploratory MANOVA

Supplementary Table S1. Exploratory MANOVA and Assumption Checks for the Comparison of the Anxiety and Control Groups (Table 2):

**Analysis**	**Outcome**	**Statistic**	Df (df1,df2)	**p-value**	**Partial η²**
**Box's M test**	Five dependent variables	Box's M = 47.493; F = 3.032	15, 62659.322	<0.001	–
**Levene's test**	BAI Total	F = 23.998	1, 128	<0.001	–
**Levene's test**	GDS Total	F = 14.436	1, 128	<0.001	–
**Levene's test**	STAI-1	F = 2.694	1, 128	0.102	–
**Levene's test**	STAI-2	F = 5.142	1, 128	0.025	–
**Levene's test**	GAS Total	F = 17.470	1, 128	<0.001	–
**MANOVA – Group effect**	**BAI, GDS, STAI-1, STAI-2 and GAS Total**	**Pillai's Trace = 0.418; F = 17.822**	**5, 124**	**<0.001**	**0.418**

An additional exploratory MANOVA was conducted using the BAI, GDS, STAI-1, STAI-2, and GAS total scores. No problematic redundancy was observed among the outcomes included in the model ($r=0.520–0.842$), and no multivariate outliers were identified based on Mahalanobis distance. Because the assumptions of homogeneity of covariance matrices and error variances were violated, Pillai's Trace was used. The overall group effect was statistically significant (Pillai's Trace=0.418, $F(5,124)=17.822$, $p<0.001$, partial $\eta^2=0.418$).

As a sensitivity analysis, square-root transformations were applied to the BAI and GAS total scores because of departures from residual normality. Following transformation, residual normality was satisfied for all outcomes (Shapiro–Wilk, all $p>0.05$), and Box's M test was non-significant ($p=0.093$). The overall group effect remained significant (Pillai's Trace=0.441, $F(5,124)=19.582$, $p<0.001$, partial $\eta^2=0.441$), supporting the robustness of the primary finding.

Supplementary Table S2. Exploratory MANOVA and Assumption Checks for Cognitive Performance in the Anxiety and Control Groups (Table 3):

Analysis	Outcome	Statistic	df (df1, df2)	p-value	Partial η^2
**Box's M test**	Six dependent variables	Box's M = 133.386; F = 6.031	21, 57308.783	<0.001	–
**Levene's test**	SMMSE Total	F = 0.016	1, 128	0.899	–
**Levene's test**	Clock Drawing Total	F = 3.399	1, 128	0.068	–
**Levene's test**	Verbal Fluency Total	F = 0.018	1, 128	0.894	–
**Levene's test**	TMT-A Time	F = 10.833	1, 128	0.001	–
**Levene's test**	TMT-B Time	F = 26.079	1, 128	<0.001	–
**Levene's test**	Enhanced Cue Recall	F = 13.280	1, 128	<0.001	–
**MANOVA – Group effect**	**SMMSE, Clock Drawing, Verbal Fluency, TMT-A, TMT-B, and Enhanced Cue Recall**	**Pillai's Trace = 0.257; F = 7.094**	**6, 123**		

An additional exploratory MANOVA was conducted using the SMMSE total score, Clock Drawing total score, Verbal Fluency total score, TMT-A and TMT-B completion times, and Enhanced Cue Recall score. Highly correlated or mathematically related scores were not simultaneously included in the model to minimize redundancy. No multivariate outliers were identified based on Mahalanobis distance. Because the assumptions of homogeneity of covariance matrices and error variances were violated, Pillai's Trace was used. The overall group effect was statistically significant (Pillai's Trace=0.257, $F(6,123)=7.094$, $p<0.001$, partial $\eta^2=0.257$).

As a sensitivity analysis, transformations were evaluated because of departures from residual normality; however, these did not fully resolve the assumption violations. Therefore, a rank-transformed sensitivity MANOVA was performed. Following rank transformation, Box's M test was non-significant ($p=0.548$), although Levene's test remained significant for TMT-B ($p=0.017$). The overall group effect remained statistically significant (Pillai's Trace=0.356, $F(6,123)=11.335$, $p<0.001$, partial $\eta^2=0.356$), supporting the robustness of the primary exploratory MANOVA finding.

Heatmap for Anxiety Group

Supplementary Figure S1A. Anxiety Group (n=70)

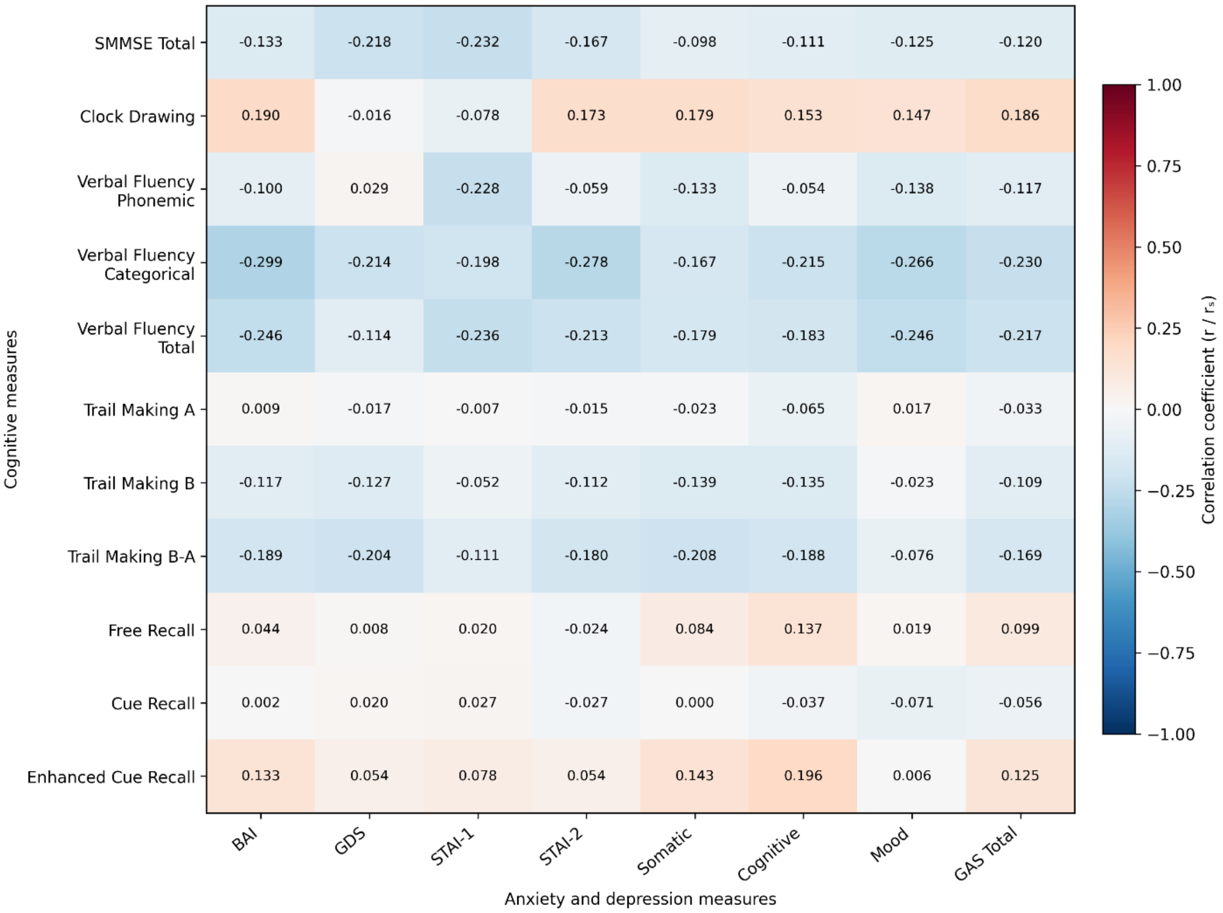

Pearson used only when both variables were normally distributed; otherwise Spearman. Bonferroni α = 0.000568. * Significant after Bonferroni correction.

Heatmap for Control Group

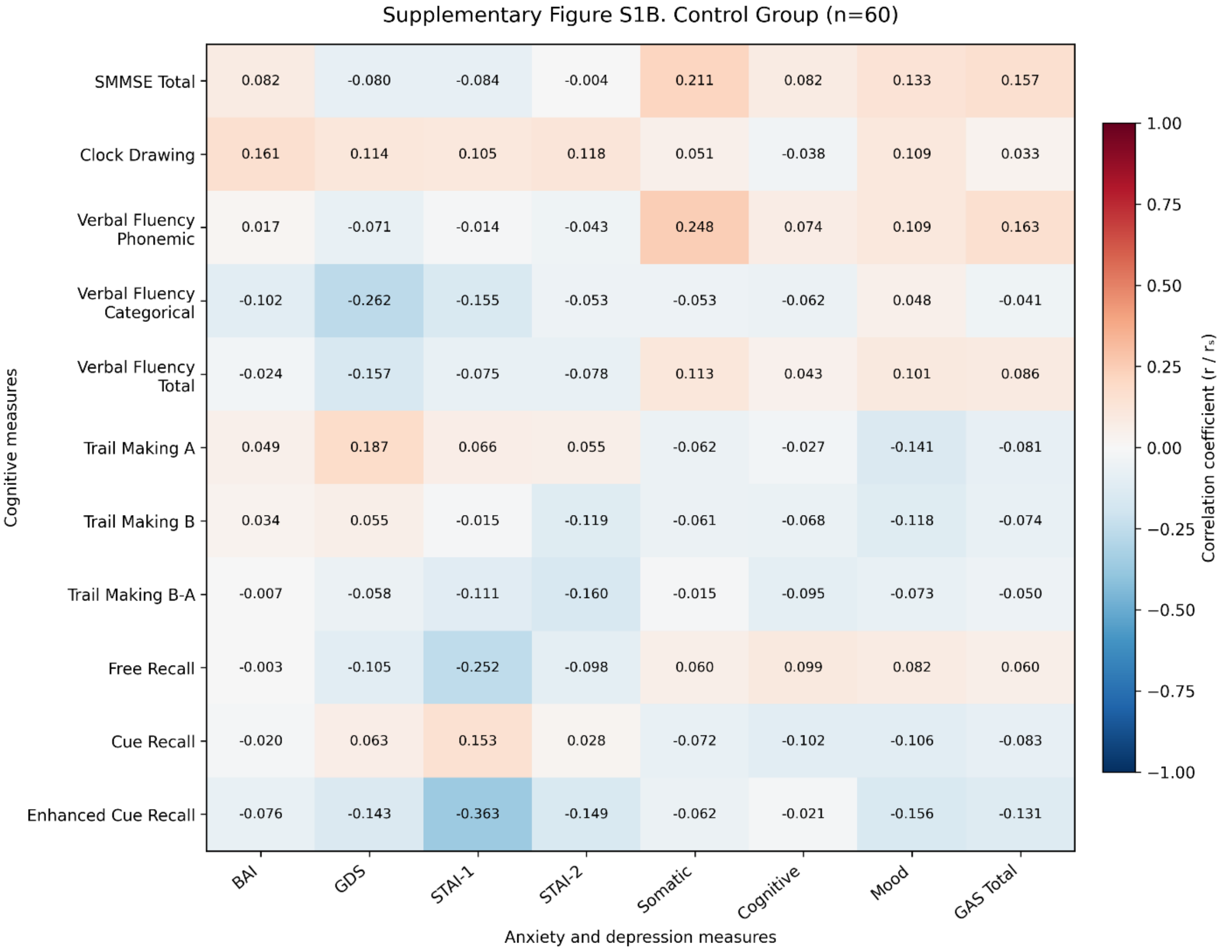

Pearson used only when both variables were normally distributed; otherwise Spearman. Bonferroni α = 0.000568. * Significant after Bonferroni correction.
